# The links of fine airborne particulate matter exposure to occurrence of cardiovascular and metabolic diseases in Michigan, USA

**DOI:** 10.1371/journal.pgph.0000707

**Published:** 2022-08-05

**Authors:** El Hussain Shamsa, Zhenfeng Song, Hyunbae Kim, Falah Shamsa, Linda D. Hazlett, Kezhong Zhang

**Affiliations:** 1 Center for Molecular Medicine & Genetics, The Wayne State University School of Medicine, Detroit, MI, United States of America; 2 Cancer Coalition of Georgia, Atlanta, GA, United States of America; 3 Ophthalmology, Visual and Anatomical Sciences, The Wayne State University School of Medicine, Detroit, MI, United States of America; 4 Department of Immunology and Microbiology, The Wayne State University School of Medicine, Detroit, MI, United States of America; University of Groningen, NETHERLANDS

## Abstract

Air pollutants, particularly airborne particulate matter with aerodynamic diameter < 2.5μm (PM_2.5_), have been linked to the increase in mortality and morbidity associated with cardiovascular and metabolic diseases. In this study, we investigated the dose-risk relationships between PM_2.5_ concentrations and occurrences of cardiovascular and metabolic diseases as well as the confounding socioeconomic factors in Michigan, USA, where PM_2.5_ levels are generally considered acceptable. Multivariate linear regression analyses were performed to investigate the relationship between health outcome and annual ground-level PM_2.5_ concentrations of 82 counties in Michigan. The analyses revelated significant linear dose-response associations between PM_2.5_ concentrations and cardiovascular disease (CVD) hospitalization. A 10 μg/m^3^ increase in PM_2.5_ exposure was found to be associated with a 3.0% increase in total CVD, 0.45% increase in Stroke, and a 0.3% increase in Hypertension hospitalization rates in Medicare beneficiaries. While the hospitalization rates of Total Stroke, Hemorrhagic Stroke, and Hypertension in urbanized counties were significantly higher than those of rural counties, the death rates of coronary heart disease and ischemic stroke in urbanized counties were significantly lower than those of rural counties. These results were correlated with the facts that PM_2.5_ levels in urbanized counties were significantly higher than that in rural counties and that the percentage of the population with health insurance and the median household income in rural counties were significantly lower. While obesity prevalence showed evidence of a weak positive correlation (*ρ* = 0.20, p-value = 0.078) with PM_2.5_ levels, there was no significant dose-response association between county diabetes prevalence rates and PM_2.5_ exposure in Michigan. In summary, this study revealed strong dose-response associations between PM_2.5_ concentrations and CVD incidence in Michigan, USA. The socioeconomic factors, such as access to healthcare resources and median household income, represent important confounding factors that could override the impact of PM_2.5_ exposure on CVD mortality.

## Introduction

Air pollution is a sustained world-wide public health concern for the general population. Accumulating evidence has linked exposure to high-levels of airborne particulate matter in fine and ultrafine ranges (aerodynamic diameter < 2.5 μm, PM_2.5_) to increased mortality and morbidity associated with cardiovascular and metabolic diseases [1–5]. There is a linear dose-risk relationship between PM_2.5_ concentrations and occurrence of cardiovascular or metabolic disease, even in the countries within guidelines for EPA PM_2.5_ exposure limits [6]. Long-term PM_2.5_ exposure increases the risk of cardiovascular, metabolic, and neurodegenerative diseases to an even greater degree and effects may be even more pronounced in susceptible sub-populations such as older adults, those with lower socioeconomic status, and individuals with pre-existing conditions. Most recently, epidemiological studies indicated that short-term exposure to polluted air, even at levels generally considered “acceptable,” may impair mental ability in elderly people in US [7]. Long-term exposure to PM_2.5_ is associated with an increased risk of anosmia, the inability to smell [8]. In developing countries, such as China, India, and Latin America, where daily and annual PM_2.5_ levels range from 100 to 200 μg/m^3^, detrimental effects of PM_2.5_ exposure on public health have been grossly underestimated. Traffic-related PM_2.5_ is a complex mixture of particles and gases from gasoline and diesel engines, together with dust from wear of road surfaces, tires, and brakes [9, 10]. Airborne PM_2.5_ exhibits an incremental capacity to penetrate to the most distal airway units and potentially the systemic circulation [11, 12]. Studies have demonstrated that these PM_2.5_ particles cause cytotoxic effects, especially upon forming complexes of PM_2.5_ particles rather than as a single or few particles [11]. Recent studies by our group and others in the field have addressed that traffic-related PM_2.5_ may promote cardiovascular and metabolic diseases, possibly by: exaggerating systemic inflammation [1, 4, 5], causing oxidative and endoplasmic reticulum (ER) stress damages [13–15], and disrupting energy homeostasis [4, 16]. While the link between PM_2.5_ pollution and occurrence of cardiovascular and metabolic diseases has been established, dose-response associations between PM_2.5_ concentrations and the subtypes of CVD or metabolic diseases, especially in the areas where PM_2.5_ levels are generally considered “acceptable”, have not been precisely defined.

In this study, we investigated the dose-risk relationships between PM_2.5_ concentrations and occurrence of cardiovascular and metabolic diseases in Michigan, USA, where the levels of PM_2.5_ are generally considered “acceptable” compared to the developing countries. Michigan is a representative state of the Midwestern region of the USA with a complex environmental and socioeconomic background, representing an ideal state for studying complex relationships between PM_2.5_ exposure and health outcome. The state of Michigan possesses a typical urban/rural county status and racial diversity as well as counties of specific health interest, such as Wayne County. Particularly, the great Detroit area possesses a number of industrial facilities and transportation routes. Residents in this area form a racially diverse community, including approximately 40% Hispanic, 27% African American, 30% White, Arabic, Native American, and others within a highly urbanized, industrial environment dominated by auto factories, steel mills, metal finishing industries, and waste processing. Our studies reveal that the levels of PM_2.5_ exposure were significantly associated with increased hospitalization rates of cardiovascular diseases (CVD), Stroke, and Hypertension in Michigan. Interestingly, while the hospitalization rate of CVD in urbanized counties was higher than that in rural counties, the death rates of CVD in rural counties were significantly higher than that in urbanized counties, implicating the impact of socioeconomic factors, such as access to healthcare resources and median household income, in conjunction with air pollution-associated mortality and morbidity.

## Resources and methods

### Data collection

Annual data was collected for county Diabetes prevalence rate from 2010 to 2016 from the CDC National Health Interview Survey (NIHS) (https://gis.cdc.gov/grasp/diabetes/DiabetesAtlas.html#). Prevalence was deemed as a more appropriate measure for rates of diabetes, as diabetes is a chronic disease treated on an outpatient basis, whereas the other diseases studied tend to be more acute events, thus hospitalization rates would not be an accurate representation of diabetes rates within Michigan. Diabetes death rate data was obtained using Michigan Department of Health and Human Services (MDHHS) yearly county mortality datasets (https://www.mdch.state.mi.us/osr/deaths/DiabetesUS.asp). All other health outcome data (hospitalizations and deaths) was retrieved from the CDC’s Division for Heart Disease and Stroke Prevention (DHDSP) (https://www.cdc.gov/dhdsp/index.htm). Based on the US Census Bureau database, 2016 Michigan population estimate is 9,950,571. The number of 2016 Michigan county Original Medicare Beneficiaries is 1,270,636. Biannual data was collected for Total Cardiovascular Disease (CVD), Coronary Heart Disease (CHD), Heart Disease (HD), Total Stroke, Ischemic Stroke, and Hemorrhagic Stroke hospitalizations and deaths as well as Hypertension hospitalizations. Death rate data was not available for both hypertension and obesity, likely because they are generally considered secondary causes of death rather than the primary cause of death. All hospitalization data was collected for Medicare Beneficiaries ages 65+ for all races and genders and was reported as Hospitalizations per 1,000 Medicare Beneficiaries; this data will be referred to as “hospitalizations” throughout the study. Death data was collected for all ages, races and genders, and was reported as Deaths per 100,000 population (all ages, races, and genders); this data will be referred to as “deaths” throughout the study. Hospitalization rate and death rate are calculated as follows:

$$Hospitalization\;Rate\;(\%)=\frac{Hospitalizations}{1,000\;Medicare\;Beneficiaries}x\;100\%$$ (1)

$$Death\;Rate\;(\%)=\frac{Deaths}{100,000\;people\;in\;county}x\;100\%$$ (2)

International Classification of Diseases, 9^th^ Revision, Clinical Modification (ICD-9-CM, for hospitalizations before 2015) and ICD-10-CM (for hospitalizations after 2015) codes for each health outcome are listed below in S1 Table. All data for the confounding variables mentioned below were also obtained from the CDC’s DHDSP. Data for 82 counties in the state of Michigan was collected. This included all Michigan counties with the exception of Keweenaw County, Michigan, which was excluded due to lack of sufficient data.

Annual concentrations of ground-level fine particulate matter (PM_2.5_, measured in μg/m^3^) using high-resolution 0.01°x0.01° gridded data retrieved from multiple satellite, surface-level monitor, and simulated data sources (S1 Fig) [17]. These gridded datasets were processed into county-wise measurements across the state of Michigan by averaging all grid values whose coordinates are contained within a respective county polygon. Coordinates for county polygons were acquired from the Homeland Infrastructure Foundation-Level Data (HIFLD) public domain (https://hifld-geoplatform.hub.arcgis.com/).

### Statistical analyses

Linear regression analysis was used to evaluate for strength of association between each health outcome and PM_2.5_ concentration. Health outcome and PM_2.5_ data were averaged over the study period, from 2010 to 2016. Two multivariate models were calculated: one with disease hospitalizations as the dependent variables and the other with disease deaths as the outcome variables. One multiple linear regression model was calculated using diabetes prevalence rate as the dependent variable. All three models were constructed using PM_2.5_ concentration as the explanatory variable of interest.

Additionally, each model was adjusted for the following 15 county-level confounding factors: obesity prevalence (ages 20+), leisure time physical inactivity (ages 20+), % black, % Hispanic, % above age 65, number of hospitals, % without health insurance, % with less than high school education (ages 25+), % poverty, median home income, county urban/rural status, % blood pressure medication nonadherence (Medicare Part D beneficiaries), % cholesterol-lowering medication nonadherence (Medicare Part D beneficiaries), % high cholesterol (among adults ages 18+ screened in 5 years), and % current smoker status (ages 18+) (Table 2). The inclusion of these confounding variables controls for lifestyle, race, age, access to healthcare, socioeconomic factors, and cardiovascular health predictors—each of which has been established to be associated with cardiovascular and metabolic disease incidence and mortality.

Given the ecological nature of the study, we calculated Moran indices (Moran I) to test for spatial autocorrelation in each measured outcome with respect to county distance. County weights used in calculating each Moran I were obtained by using an inverse distance matrix, where counties were represented as a single [longitude, latitude] point within each county polygon and gridded Euclidean distances were computed between each point [18]. The Moran I significance test was then performed to test the null hypothesis of spatial independence, and all outcome variables except diabetes prevalence showed statistically significant differences from expected values (p < 0.05), indicating significant spatial autocorrelation in these outcomes. To accommodate for this spatial autocorrelation, spatial correlation was accounted for in the parameter estimates of the two multivariate regression models, as detailed below.

Eq (3) shows the multivariate linear regression model for disease hospitalization rates:

$$\begin{array}{c} Y_{1}=\alpha_{1}+\beta_{{1,PM}_{2.5}}X_{{1,PM}_{2.5}}+\beta_{1,1}X_{1,1}+\cdots +\beta_{1,12}X_{1,12}+\varepsilon_{1}\\ \cdots \\ Y_{7}=\alpha_{7}+\beta_{{7,PM}_{2.5}}X_{{7,PM}_{2.5}}+\beta_{7,1}X_{7,1}+\cdots +\beta_{7,12}X_{7,12}+\varepsilon_{7} \end{array}$$ (3)

Where Y_1_, …, Y_7_ represent outcome variables: total CVD, CHD, HD, total stroke, ischemic stroke, hemorrhagic stroke, and hypertension hospitalization rates. Eq (4) shows the multivariate linear regression model for disease death rates:

$$\begin{array}{c} Y_{1}=\alpha_{1}+\beta_{{1,PM}_{2.5}}X_{{1,PM}_{2.5}}+\beta_{1,1}X_{1,1}+\cdots +\beta_{1,12}X_{1,12}+\varepsilon_{1}\\ \cdots \\ Y_{6}=\alpha_{6}+\beta_{{6,PM}_{2.5}}X_{{6,PM}_{2.5}}+\beta_{6,1}X_{6,1}+\cdots +\beta_{6,12}X_{6,12}+\varepsilon_{6} \end{array}$$ (4)

Where Y_1_, …, Y_6_ represent outcome variables: total CVD, CHD, HD, total stroke, ischemic stroke, and hemorrhagic stroke death rates. Eq (5) shows the multiple linear regression model for diabetes prevalence rate:

$$Y=\alpha +\beta_{{PM}_{2.5}}X_{{PM}_{2.5}}+\beta_{1}X_{1}+\cdots +\beta_{12}X_{12}+\varepsilon$$ (5)

Where Y represents the outcome variable diabetes prevalence rate. In each of the equations above, α_i_ represents the intercept, X_i, PM2.5_ represents the explanatory variable average county PM_2.5_ concentration and β_i,PM2.5_ is its respective regression coefficient, X_i,1_, …, X_i,12_ represents each of the 12 confounding variables and β_i,1_, …, β_i,12_ are their respective regression coefficients. The errors *ε*_i_ in Eqs (3) and (4) are taken to be spatially correlated with respect to county proximity (determined by using a single point within each county polygon, as described above). This correlation was computed using an exponential correlation structure, in which the correlation degrades with an exponential rate of decay with respect to distance. Regression coefficient estimates were then obtained by maximum likelihood (ML) estimation [19]. The error vector *ε* in Eq (5) was taken to consist of independently normally distributed errors, as there was no significant spatial autocorrelation in diabetes prevalence data, as described above. The normality assumption is validated with the adjustment of the random errors for spatial correlation, and the multivariate linear regression models detailed above are deemed appropriate for our data analysis. A summary of the results of the regression analyses is shown in Table 1.

**Table 1:** A summary of the results of the regression analyses. Regression coefficient (β) for PM_2.5_ is shown for each regression model, as well as the p-value for each variable. P-value < 0.05 is considered a statistically significant association. Column *a* shows results of multivariate multiple regression for hospitalization/death rates as well as multiple regression results for diabetes prevalence. Coefficients of confounding variables in the models are not shown. Colum *b* shows results of univariate simple regression with respect to each disease outcome variable, with PM_2.5_ concentration as the explanatory variable.

	Multivariate Multiple Regression^a^		Univariate Simple Regression^b^	
Health Outcome	β_PM2.5_ [95% CI]	P-value	β [95% CI]	P-value
Total CVD				
Hospitalizations	3.006 [0.760, 5.252]	0.011	3.364 (1.959, 4.769)	< 0.001
Deaths	4.537 [0.803, 8.271]	0.020	2.472 (-0.802, 5.746)	0.137
Coronary Heart Disease				
Hospitalizations	1.039 [0.267, 1.812]	0.010	0.502 (-0.009, 1.013)	0.054
Deaths	1.693 [-1.596, 4.982]	0.317	-0.496 (-3.085, 2.094)	0.704
Heart Disease				
Hospitalizations	2.604 [0.662, 4.547]	0.011	2.760 (1.561, 3.959)	< 0.001
Deaths	3.382 [-0.399, 7.163]	0.084	1.997 (-1.086, 5.080)	0.201
Total Stroke				
Hospitalizations	0.448 [0.197, 0.698]	< 0.001	0.526 (0.380, 0.671)	< 0.001
Deaths	0.078 [-0.697, 0.854]	0.844	-0.234 (-0.668, 0.199)	0.285
Ischemic Stroke				
Hospitalizations	0.371 [0.143, 0.598]	0.002	0.368 (0.237, 0.499)	< 0.001
Deaths	0.331 [-0.733, 0.072]	0.112	-0.373 (-0.601, -0.146)	0.002
Hemorrhagic Stroke				
Hospitalizations	0.039 [0.014, 0.065]	0.004	0.071 (0.057, 0.084)	< 0.001
Deaths	0.084 [-0.068, 0.235]	0.283	0.005 (-0.075, 0.084)	0.908
Hypertension Hospitalizations	0.290 (0.042, 0.538)	0.025	0.525 (0.370, 0.679)	< 0.001
Diabetes Prevalence	-0.095 (-0.214, 0.023)	0.119	0.012 (-0.085, 0.110)	0.801

Table 2 shows descriptive statistics for each of the variables included in the study for all 82 counties (Total), as well as for urban and rural counties. Non-parametric Wilcoxon Rank Sum tests were used in comparing means of urban vs. rural counties. P-values < 0.05 are considered statistically significant in this study. Lastly, Spearman’s correlation coefficients were calculated between all variables and are shown as a correlation matrix in S2 Table. All statistical analyses in this study were performed using R (version 3.6.2).

**Table 2:** Characteristics of Michigan counties used in the study. Mean (standard deviation), Minimum County value, and Maximum County value for all counties (Total), counties with urban status (Urban), and counties with rural or non-urban status (Rural). Fold is the ratio of urban and rural means, equal to (Urban Mean)/(Rural Mean).P-values obtained from Wilcoxon Rank Sum Tests between Urban and Rural County values are shown. P-value < 0.05 is statistically significant.

Outcome	Total (n = 82 counties)			Urban (n = 26 counties)	Rural (n = 56 counties)		
	Mean (SD)	Min	Max	Mean (SD)	Mean (SD)	Fold	P-value
Total CVD							
Hospitalization Rate	70.08 (14.50)	38.83	103.93	75.10 (13.62)	67.75 (14.42)	1.108	0.088
Death Rate	246.7 (30.23)	162.6	318.2	243.4 (31.78)	248.3 (29.65)	0.980	0.417
Coronary Heart Disease							
Hospitalization Rate	17.55 (4.76)	10.23	32.57	17.21 (5.24)	17.70 (4.56)	0.972	0.449
Death Rate	127.3 (23.61)	75.7	176.43	118.41 (23.67)	131.5 (22.61)	0.900	0.008
Heart Disease							
Hospitalization Rate	52.39 (12.27)	27.87	85.17	56.35 (11.68)	50.55 (12.19)	1.115	0.088
Death Rate	193.6 (28.37)	119.9	258.4	189.8 (28.49)	195.3 (28.40)	0.972	0.302
Total Stroke							
Hospitalization Rate	11.60 (1.70)	7.28	16.10	12.54 (1.38)	11.16 (1.66)	1.124	0.002
Death Rate	38.42 (3.98)	30.97	52.57	37.73 (4.83)	38.75 (3.51)	0.974	0.062
Ischemic Stroke							
Hospitalization Rate	9.40 (1.41)	5.90	12.37	9.95 (1.11)	9.15 (1.47)	1.087	0.051
Death Rate	20.47 (2.21)	16.47	29.63	19.48 (1.83)	20.93 (2.23)	0.931	0.005
Hemorrhagic Stroke							
Hospitalization Rate	1.39 (0.19)	0.90	2.00	1.55 (0.13)	1.32 (0.17)	1.174	< 0.001
Death Rate	9.20 (0.72)	7.57	11.53	9.20 (0.84)	9.20 (0.67)	1.000	0.799
Hypertension Hosp. Rate	2.89 (1.76)	0.83	10.17	4.25 (2.30)	2.25 (0.95)	1.889	< 0.001
Diabetes Prevalence	9.39 (0.89)	7.47	11.14	9.38 (1.03)	9.40 (0.82)	0.998	0.972
PM_2.5_ (μg/m^3^)	8.14 (2.03)	4.94	12.08	10.15 (0.90)	7.21 (1.70)	1.408	< 0.001
Obesity (%)	33.38 (4.33)	23.40	43.10	33.32 (4.15)	33.41 (4.45)	0.997	0.996
Leisure Physical Inactivity (%)	23.33 (3.65)	13.90	30.30	22.86 (3.12)	23.55 (3.89)	0.971	0.431
Black (%)	3.82 (5.96)	0.10	38.70	8.40 (8.47)	1.69 (2.25)	4.970	< 0.001
Hispanic (%)	3.54 (2.58)	1.00	14.80	5.11 (2.45)	2.81 (2.32)	1.819	< 0.001
White (%)	87.72 (8.91)	49.50	96.00	81.13 (10.54)	90.79 (6.03)	0.894	< 0.001
Age above 65 (%)	20.54 (5.13)	11.70	35.50	16.37 (1.87)	22.48 (5.01)	0.728	< 0.001
# Hospitals	1.56 (1.98)	0	14	2.81 (3.06)	0.98 (0.67)	2.867	< 0.001
No Health Insurance (%)	6.92 (1.27)	4.10	11.50	5.90 (0.96)	7.39 (1.12)	0.798	< 0.001
Less than High School Education (%)	9.64 (2.83)	4.60	17.00	8.79 (2.51)	10.03 (2.90)	0.876	0.051
Poverty (%)	13.92 (3.68)	5.00	23.40	12.93 (4.11)	14.38 (3.41)	0.899	0.19
Median Household Income	51,561 (9,501)	36,000	84,000	59,346 (10,107)	47,946 (6,675)	1.238	< 0.001
Median Home Value	125,585 (37,805)	68,000	256,000	147,192 (40,165)	115,554 (32,380)	1.274	< 0.001
High cholesterol (%)	38.45 (3.29)	30.00	45.30	35.98 (2.38)	39.59 (3.02)	0.909	< 0.001
Cholesterol Med Nonadherence (%)	14.55 (1.23)	12.20	20.20	15.41 (1.58)	14.14 (0.77)	1.090	< 0.001
Blood Pressure Med Nonadherence (%)	19.08 (1.26)	16.50	25.30	19.91 (1.75)	18.69 (0.69)	1.065	< 0.001
Current Smokers (%)	21.06 (2.45)	13.90	26.20	19.80 (2.71)	21.65 (2.10)	0.915	0.004

## Results

### A dose-response association between PM_2.5_ exposure and hospitalization of cardiovascular disease, stroke, and hypertension, but not diabetes, exists in the state of Michigan

**Fig 1:**
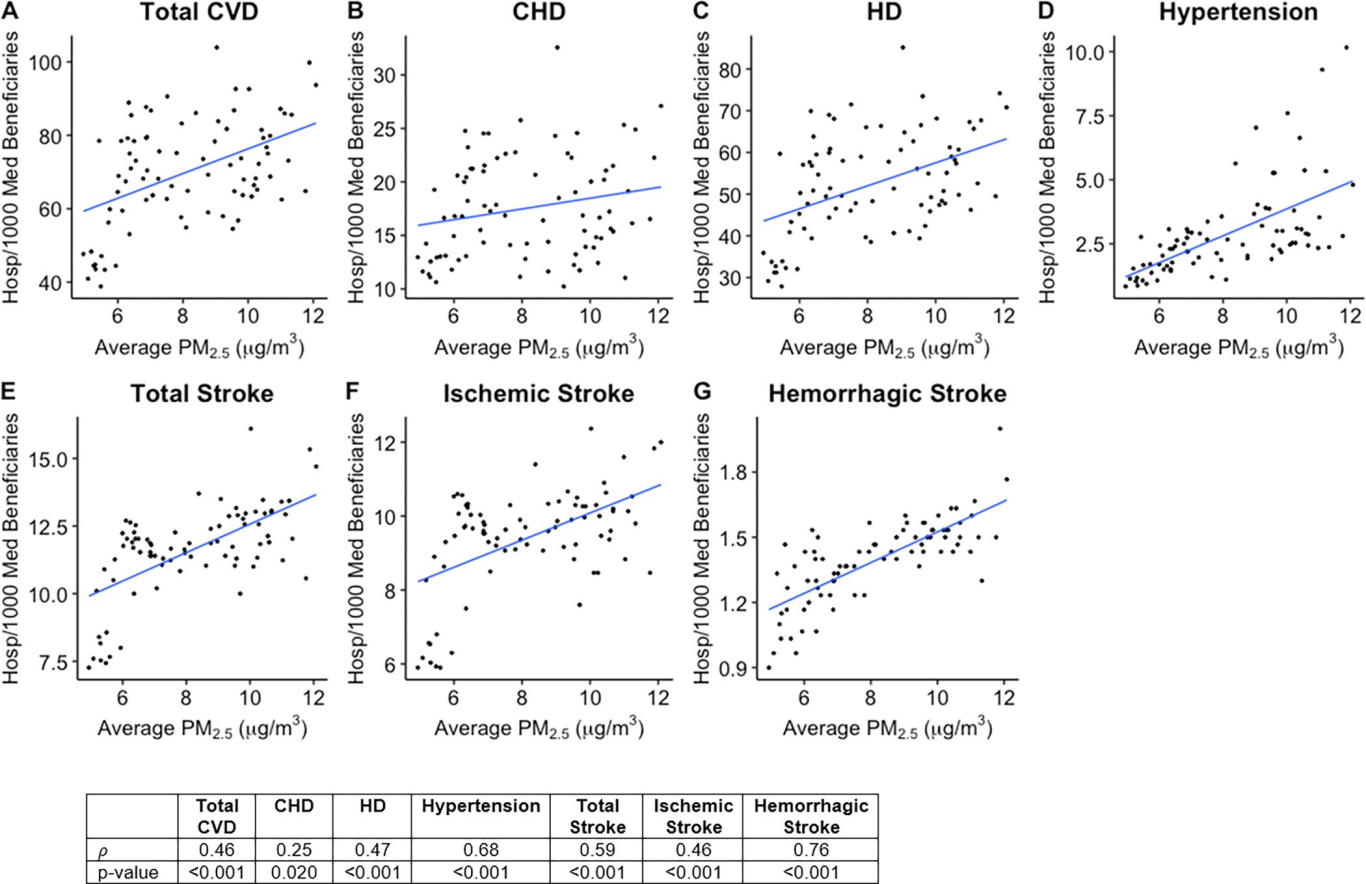
Scatterplots showing average PM_2.5_ (x axis) vs. Hospitalizations (y axis) for: **A.** Total Cardiovascular Disease (CVD), **B.** Coronary Heart Disease (CHD), **C.** Heart Disease (HD), **D.** Hypertension, **E.** Total Stroke, **F.** Ischemic Stroke, **G.** Hemorrhagic Stroke. Hospitalizations have units: Disease hospitalizations per 1000 Medicare beneficiaries (ages 65+) within the county. Individual points represent average values for each of the 82 counties included in the study. Best fit line for each plot was obtained by simple linear regression. Table shows Spearman rank correlation coefficient (*ρ*) calculated for each plot as well as the p-value for each correlation coefficient. P-values < 0.05 are statistically significant.

**Fig 2:**
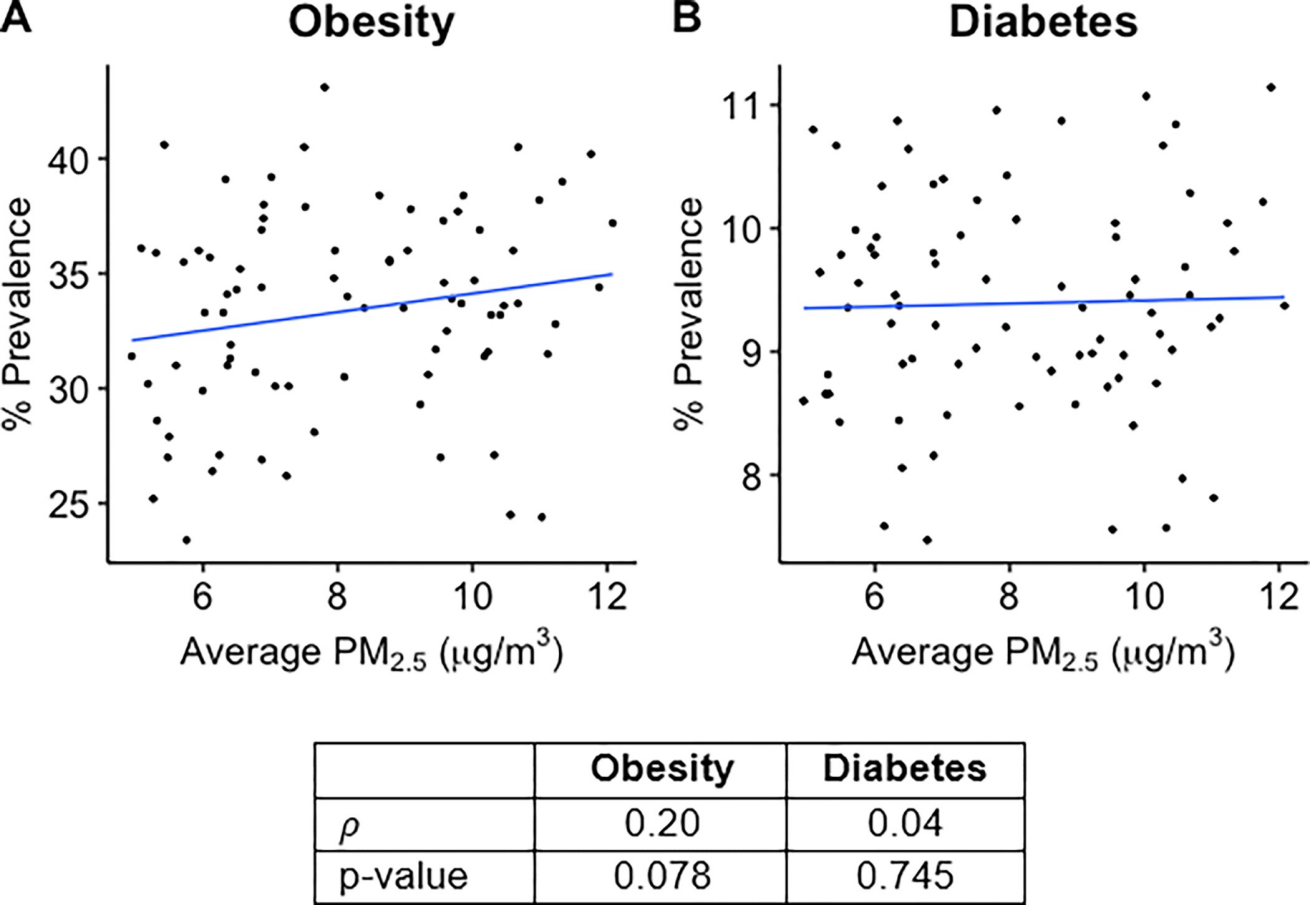
Scatterplots showing average PM_2.5_ (x axis) vs. Prevalence Rate (y axis) for: **A.** Diabetes and **B.** Obesity. Prevalence Rates are shown as a percentage value. Individual points represent average values for each of the 82 counties included in the study. Best fit line for each plot was obtained by simple linear regression. Table shows Spearman rank correlation coefficient (*ρ*) calculated for each plot as well as the p-value for each correlation coefficient. P-values < 0.05 are statistically significant.

**Fig 3:**
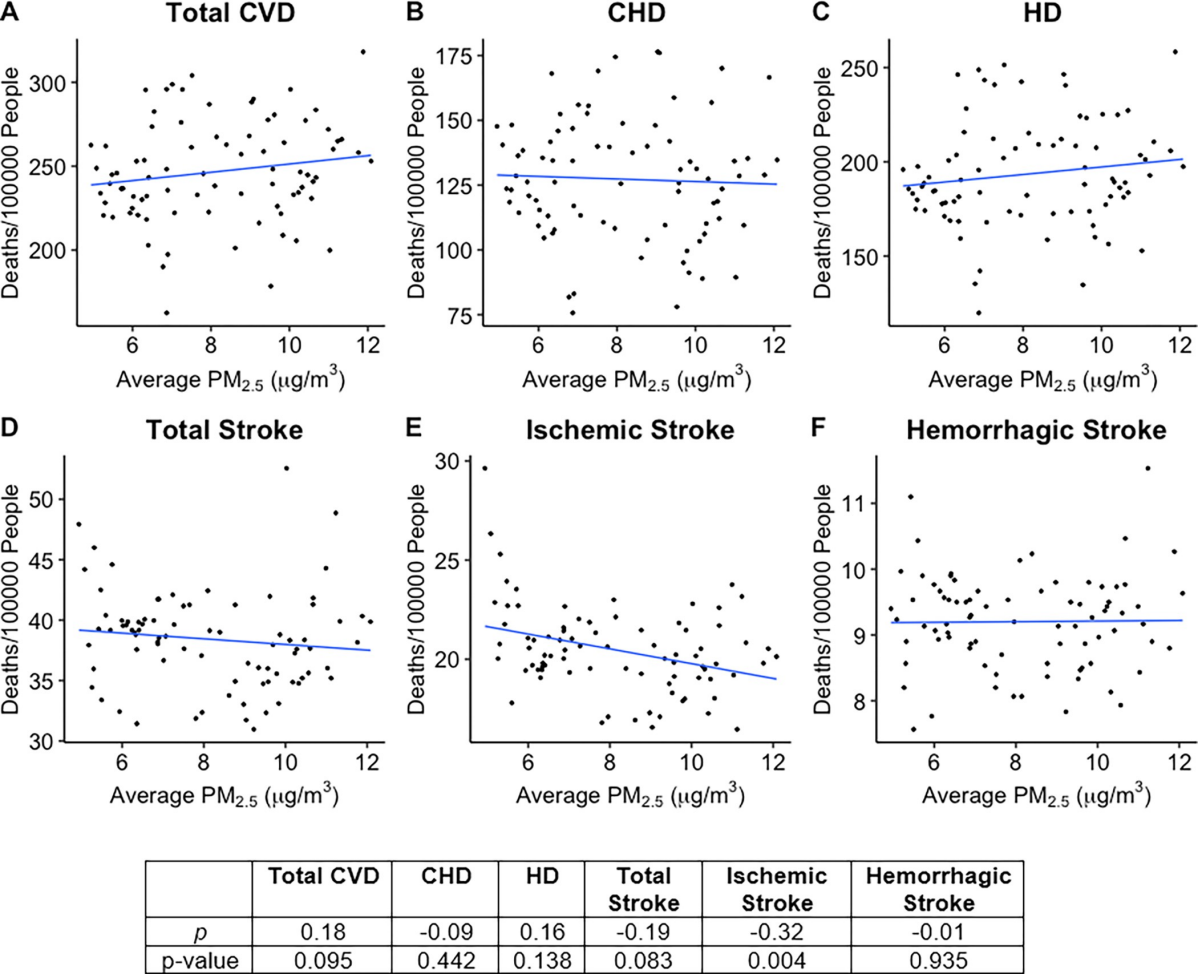
Scatterplots showing average PM_2.5_ vs. death rate **A.** Total Cardiovascular Disease (CVD), **B.** Coronary Heart Disease (CHD), **C.** Heart Disease (HD), **D.** Total Stroke, **E.** Ischemic Stroke, and **F.** Hemorrhagic Stroke. Deaths have units: Disease deaths per 100,000 people within the county, regardless of ages or Medicare status (Y axis). Individual points represent values for each of the 82 counties included in the study. Best fit line for each plot obtained by simple linear regression. Table shows Spearman rank correlation coefficient (*ρ*) calculated for each plot as well as the p-value for each correlation coefficient. P-values < 0.05 are statistically significant.

Health outcome data for each of the 82 Michigan counties studied was plotted against average county PM_2.5_ concentration, and Spearman’s correlation coefficient (*ρ*) was calculated for each plot (Figs 1–3). As shown in Fig 1, Total Cardiovascular Disease (CVD, Spearman’s *ρ* = 0.46), Coronary Heart Disease (CHD, *ρ* = 0.25), Heart Disease (HD, *ρ* = 0.47), Total Stroke (*ρ* = 0.59), Ischemic Stroke (*ρ* = 0.46), Hemorrhagic Stroke (*ρ* = 0.76), and Hypertension (HTN, *ρ* = 0.68) hospitalizations have strong positive correlations with PM_2.5_ concentrations over the 82 counties studied. Unlike the hospitalizations, deaths of cardiovascular disease did not appear to be significantly correlated with PM_2.5_ levels in Michigan (Fig 3). Of note, however, total CVD deaths have a weak positive correlation with average PM_2.5_ concentration (*ρ* = 0.18). Interestingly, Ischemic stroke was found to have a strongly negative correlation (*ρ* = -0.32) while Total Stroke had a weak negative correlation (*ρ* = -0.19) with average PM_2.5_ concentration. Studies have linked PM_2.5_ exposure to increased incidences of metabolic syndrome, particularly diabetes mellitus [20–23]. However, diabetes prevalence rates showed no correlation (*ρ* = 0.04) with county PM_2.5_ concentrations in Michigan, while obesity prevalence rates did show a weak positive correlation (*ρ* = 0.20, p-value = 0.078) with county PM_2.5_ concentrations, though it was not statistically significant (Fig 2).

We performed multivariate multiple linear regression analyses to assess for associations between ground-level PM_2.5_ concentrations and county health outcome data for the years 2010 to 2016. These models were adjusted for major health determinants that are well-known to be linked to cardiovascular, cerebrovascular, and metabolic diseases, such as obesity and sedentary lifestyle, race, socioeconomic status, access to healthcare, hyperlipidemia, and medication nonadherence. Adjusting for these factors allows for correction of the concurrent impact of confounding factors, as well as comparison with simple linear regression results to analyze the degree of distortion caused by these factors. As shown in Table 1, all cardiovascular disease and cerebrovascular disease hospitalizations were found to be significantly positively associated with PM_2.5_ when accounting for the confounding factors. For each 1 μg/m^3^ increase in PM_2.5_ concentration, it was found that total CVD hospitalizations increased by 3.006 hospitalizations per 1,000 Medicare Beneficiaries (3.01% increase in hospitalization rate per 10 μg/m^3^, p-value = 0.011), while cardiovascular disease subsets HD hospitalizations and CHD hospitalizations increase by 1.039 (1.04% increase in hospitalization rate per 10 μg/m^3^, p-value = 0.010) and 2.604 (2.64% increase in hospitalization rate per 10 μg/m^3^, p-value = 0.011), respectively. Furthermore, a 1 unit increase in PM_2.5_ concentration was also found to be associated with an increase in the hospitalizations of total stroke and its subsets ischemic stroke and hemorrhagic stroke by 0.448 (0.45% increase per 10 μg/m^3^, p-value < 0.001), 0.371 (0.37% increase per 10 μg/m^3^, p-value = 0.002), and 0.039 (0.04% increase per 10 μg/m^3^, p-value = 0.004) hospitalizations per 1,000 Medicare Beneficiaries, respectively. Hypertension hospitalizations were also found to increase by 0.290 for each 1 μg/m^3^ increase in PM_2.5_ concentration (0.29% increase per 10 μg/m^3^, p-value = 0.025). Diabetes prevalence rate had no significant association with PM_2.5_ concentration.

Of the diseases studied, only Total CVD death rates were found to be significantly associated with PM_2.5_ concentration, while mortality rates of CVD subsets CHD and HD and all stroke subsets showed no significant association with PM_2.5_ when adjusting for confounding factors (Table 1, Fig 3). A 1 μg/m^3^ increase in county PM_2.5_ concentration was associated with an increase in Total CVD deaths by 4.537 per 100,000 people (0.05% increase in death rate per 10 μg/m^3^, p-value = 0.020) (Table 1). Heart disease mortality rates showed some association with average county PM_2.5_ (β = 3.382, p-value = 0.084), but this association was not statistically significant. Though Ischemic Stroke deaths were found to have a significant negative association by simple linear regression (p-value = 0.002), this association disappeared once the model was adjusted for confounding factors (p-value = 0.659). This indicates that the strong negative correlation between Ischemic Stroke deaths and PM_2.5_ concentration (Fig 3) noted above is likely due to interactions between PM_2.5_ concentration and confounding factors resulting in a potentially misleading apparent association between PM_2.5_ and Ischemic stroke mortality. This was also seen with diabetes deaths, where simple linear regression showed a significant negative association (p-value = 6.64 x 10^−6^) which disappeared in the multiple linear regression model which adjusted for confounding factors (p-value = 0.807) (Table 1).

### Urbanized counties showed significantly higher PM_2.5_ concentrations and hospitalization rates of stroke and hypertension than rural counties

**Fig 4:**
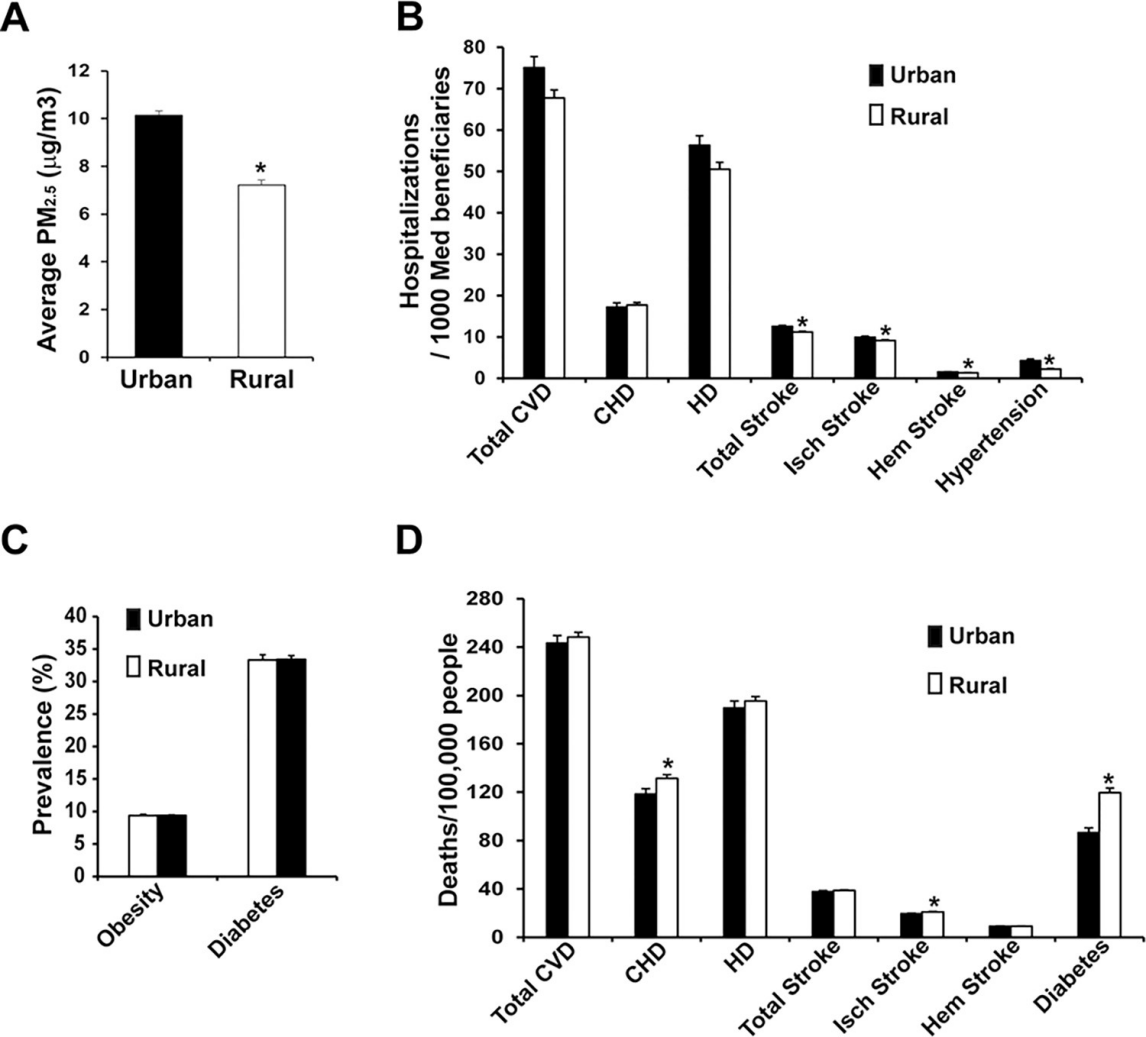
Bar graphs comparing average urban vs. rural county values for **A.** PM_2.5_ concentration, **B.** Disease Hospitalization Rate, **C.** Prevalence rate for obesity and diabetes, and **D.** Disease Death Rate. * represents a statistically significant (p-value < 0.05) difference between urban and rural values. Error bars depict standard error.

We compared the hospitalization and death rates of cardiovascular and metabolic disease between urbanized and rural counties in Michigan. As shown in Fig 4 and Table 2, urban counties were found to have significantly higher PM_2.5_ levels than rural counties (p-value < 0.001). Disease hospitalization rates for Total CVD (p-value = 0.088), HD (p-value = 0.088), Total Stroke (p-value = 0.002), Ischemic Stroke (p-value = 0.051), Hemorrhagic Stroke (p-value < 0.001), and Hypertension (p-value = 0.008) in urban counties were higher than those in rural counties, while CHD hospitalization rates and diabetes prevalence rates were similar in urban vs. rural counties. Opposite to hospitalization rates, CHD, Total Stroke, and Ischemic Stroke death rates were significantly lower in the urban counties, compared to those in the rural counties (p-values = 0.008, 0.062, and 0.005, respectively) (Fig 4, Table 2).

We compared values of various socioeconomic factors in urban versus rural counties to further unveil possible explanations for the above-mentioned trends in hospitalization and death rates in urban compared to rural counties. Percent Black and Hispanic populations (p-values < 0.001) were significantly larger in urban compared to rural counties. Interestingly, though important population-level cardiovascular disease predictors, such as elderly age (above 65), high cholesterol, and current smoker percentages, were significantly higher in rural counties, cholesterol lowering medication and blood pressure medication nonadherence were significantly higher in urban counties (all p-values < 0.001). The number of hospitals on average (p-value < 0.001), percentage of the population with health insurance (p-value < 0.001), and median household income (p-value < 0.001) were all significantly higher in urban counties compared to rural counties (Table 2), indicating that residents of urban counties in Michigan had better access to healthcare as well as greater resources to seek medical aid with.

## Discussion

In this retrospective study, we confirmed the link between PM_2.5_ concentrations and CVD and cerebrovascular diseases and made significant expansions on past studies to show strong dose-response links between PM_2.5_ concentrations and CVD subtypes, Ischemic Stoke, Hemorrhagic Stroke, and Hypertension in Michigan, USA, where the levels of PM_2.5_ are generally considered “acceptable.” While the EPA’s annual standard for PM_2.5_ levels is 12 μg/m^3^ [24], the counties we analyzed had, on average over the study period, a mean PM_2.5_ level of 8.14 μg/m^3^ and a maximum of 12.08 μg/m^3^ (Table 1). We demonstrated a strong and significant dose-response association, in which the level of PM_2.5_ exposure is positively associated with the occurrence of both CVD and cerebrovascular diseases, despite the fact that Michigan counties were on average within the EPA standard for PM_2.5_. Thus, though levels below this range may be less harmful, they are still significantly associated with disease. This is a significant finding, as it not only highlights the dramatic health consequences even small levels of PM_2.5_ may carry. The strong dose-response links between PM_2.5_ concentrations and Ischemic Stroke, Hemorrhagic Stroke, and Hypertension, among the CVD subtypes, are significant, as these associations have not been precisely defined in the past.

The state of Michigan has a complex urban/rural county status and diverse communities with variable healthcare resources and household income. Michigan represents an ideal place to study the complex impact of environmental and socioeconomic factors in public health. We found that percent Black and Hispanic populations are significantly higher in urban compared to rural counties in Michigan. Furthermore, we found that cholesterol and blood pressure controlling medication nonadherence rates were significantly higher in urban counties. Black and Hispanic races and medication nonadherence have been well documented to be associated with worsened cardiovascular health outcomes. This is further evidenced by our findings of their highly positive correlations with disease hospitalization rates in our study (S2 Table). Though we note that these urban risk factors are directly opposed by the significantly higher percentages of elderly aged residents, percentages of current smokers, and prevalence of high cholesterol in the rural counties, it is arguable that the urban risk factors were seen to have much stronger correlations with hospitalization rates (S2 Table). Thus, in addition to the strong positive associations we have shown between average county PM_2.5_ levels and cardiovascular and cerebrovascular disease hospitalization rates, the factors of race and medication nonadherence may provide a partial explanation to our findings that the hospitalization rates of cardiovascular and cerebrovascular diseases were significantly higher in urban compared to rural counties.

Importantly, our studies revealed that the associations between disease death rates and PM_2.5_ exposure may not be consistent with the associations seen between hospitalization rates and PM_2.5_ among these diverse populations. This was especially seen when observing the strong negative correlation between Ischemic Stroke deaths and PM_2.5_ concentration. Though Ischemic Stroke death rate showed a significant negative correlation with PM_2.5_ concentration, regression analysis adjusted for confounders showed no association between the two variables. Thus, possible explanation for the negative correlation seen is that counties with higher PM_2.5_ tend to be more developed. We used urban status as a measure of the degree of development and found that PM_2.5_ levels were significantly higher while Ischemic Stroke death rates were significantly lower in urbanized counties compared to rural counties. Furthermore, the number of hospitals on average, the percentage of the population with health insurance, and median household income were significantly higher in urban counties; thus, urban counties have more healthcare resources and greater access to healthcare than rural counties. As timely medical attention is critical to Ischemic Stroke survival, this is a likely explanation for the significantly lower death rates of Ischemic Stroke in urban counties, thus resulting in the observed negative correlation between Ischemic Stroke death rates and PM_2.5_ concentrations. Simple linear regression did show a strong negative association between Ischemic Stroke deaths and average PM_2.5_ concentration, however this association completely disappeared in the multivariate multiple linear regression model which corrected for confounding factors–this is further evidence that the correlation between Ischemic Stroke and PM_2.5_ levels is due to distortion by confounding factors, such as access to healthcare. Similar reasoning can also be applied to the other diseases studied. While the hospitalization rates for total CVD, HD, total stroke, hemorrhagic stroke, and hypertension were significantly higher in urban counties, the death rates of CHD and total stroke were all significantly lower in urban counties than in rural counties. This, again can be attributed to the decreased access to healthcare resources in rural counties. This observation suggests that the access to healthcare resources and household income could be important factors that mitigate the impact of environmental factors, such as PM_2.5_ exposure, in CVD-associated mortality.

It has been reported that PM_2.5_ exposure is associated with the prevalence of metabolic syndrome, particularly diabetes mellitus [20–23]. However, our studies showed that PM_2.5_ may not be associated with the prevalence of diabetes in a state where the link between PM_2.5_ and CVD and obesity was confirmed. The complex factors involving this discrepancy remains to be elucidated in future studies.

The conclusion that PM_2.5_ is a causative risk factor for CVD is consistent with the previous studies with the populations in Asia [25, 26], Europe [27, 28], and USA [29, 30]. The strengths of our study include a large sample size, investigation of CVD subtypes, and adjustment for and analysis of the effects of multiple confounding factors, including obesity, race, health care resources, household income, and variables directly linked to cardiovascular health, such as elderly age, smoking, high cholesterol prevalence, and blood pressure and cholesterol controlling medication nonadherence. A multivariate multiple linear regression model was used to assess for associations between health outcomes and PM_2.5_ concentrations while adjusting for these confounders. Some limitations are present in our study. It is a retrospective, CDC data-based study drawn from survey of public domain but not from the community. A potential bias in case-control studies is discerning the temporal relationship between risk factors and clinical outcomes, because of the complex relationships between PM_2.5_ exposure and disease occurrence. Additionally, the assessment of some outcomes and covariates relies on self-reporting that may involve unknown misclassifications. Individual variations in long-term exposures to PM_2.5_ are not trackable. Due to the nature of self-reporting and the dependency on the existing state records, we were limited in addressing the issues of unknown misclassification and individual variations. Nevertheless, to circumvent the weaknesses related to our study, we conducted the analysis with multiple CVD-related risk factors and demographics as well as socioeconomic confounding factors. Indeed, the positive correlation between risk factors and CVD indicated by our analyses were consistent with the established conclusions [31]. This validated the accuracy of our analyses, despite the use of retrospective data, in predicting the relationship between PM_2.5_ concentrations and cardiovascular and metabolic diseases.

## Conclusion

The hospitalization rates of Total CVD, Stroke and Hypertension are significantly associated with the levels of PM_2.5_ exposure in Michigan, USA, where the air quality is generally considered as acceptable. While the hospitalization rates of CVD, Stroke, and Hypertension in rural counties were significantly lower than those in urbanized counties, the death rates of CVD and Stroke subtypes were significantly higher in rural counties, partially attributed to the decreased access to healthcare resources and lower median household income. These findings indicate that the socioeconomic factors, such as access to healthcare resources and median household income, are significantly associated with the incidence of air pollution-associated mortality and morbidity.

The findings of the current study indicate that PM_2.5_ exposure in the USA is an important and major risk factor for both CVD and cerebrovascular disease incidence and mortality. Development of specific guidelines for the prevention and treatment of CVD and cerebrovascular disease needs to consider these findings. Both control of PM_2.5_ levels and improvement in healthcare resources should be warranted. Such guidelines should outline appropriate and safe treatment to prevent CVD and cerebrovascular disease in patient populations that are at increased risk due to levels of PM_2.5_ exposure, with the ultimate goal of preventing progressive disease development within the population.

## Supporting information

S1 Fig Heat maps showing PM_2.5_ levels across the state of Michigan for years 2010–2016. (DOCX)

S1 Table Clinical modification codes for cardiovascular and cerebrovascular diseases. (DOCX)

S2 Table Correlation matrix showing Spearman correlation coefficients (*ρ*) between all variables. (DOCX)

## References

[1] Sun Q, Yue P, Deiuliis JA, Lumeng CN, Kampfrath T, Mikolaj MB, et al. Ambient Air Pollution Exaggerates Adipose Inflammation and Insulin Resistance in a Mouse Model of Diet-Induced Obesity. Circulation. 2009. Epub 2009/01/21. CIRCULATIONAHA.108.799015 [pii] doi: 10.1161/CIRCULATIONAHA.108.799015 . 19153269 PMC3845676

[2] Wieckowska A, McCullough AJ, Feldstein AE. Noninvasive diagnosis and monitoring of nonalcoholic steatohepatitis: present and future. Hepatology. 2007;46(2):582–9. Epub 2007/07/31. doi: 10.1002/hep.21768 . 17661414

[3] Birkenfeld AL, Shulman GI. Non alcoholic fatty liver disease, hepatic insulin resistance and type 2 diabetes. Hepatology. 2013. Epub 2013/08/10. doi: 10.1002/hep.26672 . 23929732 PMC3946772

[4] Zheng Z, Xu X, Zhang X, Wang A, Zhang C, Huttemann M, et al. Exposure to ambient particulate matter induces a NASH-like phenotype and impairs hepatic glucose metabolism in an animal model. Journal of hepatology. 2013;58(1):148–54. Epub 2012/08/21. doi: 10.1016/j.jhep.2012.08.009 ; PubMed Central PMCID: PMC3527686. 22902548 PMC3527686

[5] Zheng Z, Zhang X, Wang J, Dandekar A, Kim H, Qiu Y, et al. Exposure to fine airborne particulate matters induces hepatic fibrosis in murine models. Journal of hepatology. 2015. doi: 10.1016/j.jhep.2015.07.020 . 26220751 PMC5003300

[6] Pearson JF, Bachireddy C, Shyamprasad S, Goldfine AB, Brownstein JS. Association between fine particulate matter and diabetes prevalence in the U.S. Diabetes care. 2010;33(10):2196–201. Epub 2010/07/16. doi: 10.2337/dc10-0698 ; PubMed Central PMCID: PMC2945160. 20628090 PMC2945160

[7] Gao X, Coull B, Lin X, Vokonas P, Spiro A, Hou L, et al. Short-term air pollution, cognitive performance and nonsteroidal anti-inflammatory drug use in the Veterans Affairs Normative Aging Study. Nature Aging. 2021;1(5):430–7. doi: 10.1038/s43587-021-00060-4 34841262 PMC8622756

[8] Zhang Z, Rowan NR, Pinto JM, London NR, Lane AP, Biswal S, et al. Exposure to Particulate Matter Air Pollution and Anosmia. JAMA Netw Open. 2021;4(5):e2111606. Epub 2021/05/28. doi: 10.1001/jamanetworkopen.2021.11606 ; PubMed Central PMCID: PMC8160589. 34042992 PMC8160589

[9] Alfaro-Moreno E, Martinez L, Garcia-Cuellar C, Bonner JC, Murray JC, Rosas I, et al. Biologic effects induced in vitro by PM10 from three different zones of Mexico City. Environ Health Perspect. 2002;110(7):715–20. Epub 2002/07/16. sc271_5_1835 [pii]. doi: 10.1289/ehp.02110715 . 12117649 PMC1240918

[10] Soukup JM, Becker S. Human alveolar macrophage responses to air pollution particulates are associated with insoluble components of coarse material, including particulate endotoxin. Toxicol Appl Pharmacol. 2001;171(1):20–6. Epub 2001/02/22. doi: 10.1006/taap.2000.9096 S0041-008X(00)99096-3 [pii]. . 11181108

[11] Brook RD, Franklin B, Cascio W, Hong Y, Howard G, Lipsett M, et al. Air pollution and cardiovascular disease: a statement for healthcare professionals from the Expert Panel on Population and Prevention Science of the American Heart Association. Circulation. 2004;109(21):2655–71. Epub 2004/06/03. doi: 10.1161/01.CIR.0000128587.30041.C8 109/21/2655 [pii]. . 15173049

[12] Nemmar A, Hoylaerts MF, Hoet PH, Dinsdale D, Smith T, Xu H, et al. Ultrafine particles affect experimental thrombosis in an in vivo hamster model. Am J Respir Crit Care Med. 2002;166(7):998–1004. Epub 2002/10/03. doi: 10.1164/rccm.200110-026OC . 12359661

[13] Sun Q, Yue P, Ying Z, Cardounel AJ, Brook RD, Devlin R, et al. Air pollution exposure potentiates hypertension through reactive oxygen species-mediated activation of Rho/ROCK. Arterioscler Thromb Vasc Biol. 2008;28(10):1760–6. Epub 2008/07/05. ATVBAHA.108.166967 [pii] doi: 10.1161/ATVBAHA.108.166967 . 18599801 PMC2739008

[14] Folkmann JK, Risom L, Hansen CS, Loft S, Moller P. Oxidatively damaged DNA and inflammation in the liver of dyslipidemic ApoE-/- mice exposed to diesel exhaust particles. Toxicology. 2007;237(1–3):134–44. Epub 2007/07/03. S0300-483X(07)00266-1 [pii] doi: 10.1016/j.tox.2007.05.009 . 17602821

[15] Laing S, Wang G, Briazova T, Zhang C, Wang A, Zheng Z, et al. Airborne particulate matter selectively activates endoplasmic reticulum stress response in the lung and liver tissues. American journal of physiology Cell physiology. 2010;299(4):C736–49. Epub 2010/06/18. doi: 10.1152/ajpcell.00529.2009 ; PubMed Central PMCID: PMC2957267. 20554909 PMC2957267

[16] Xu X, Liu C, Xu Z, Tzan K, Zhong M, Wang A, et al. Long-term Exposure to Ambient Fine Particulate Pollution Induces Insulin Resistance and Mitochondrial Alteration in Adipose Tissue. Toxicological sciences: an official journal of the Society of Toxicology. 2011;124(1):88–98. Epub 2011/08/30. doi: 10.1093/toxsci/kfr211 ; PubMed Central PMCID: PMC3196653. 21873646 PMC3196653

[17] van Donkelaar A, Martin RV, Brauer M, Hsu NC, Kahn RA, Levy RC, et al. Global Estimates of Fine Particulate Matter using a Combined Geophysical-Statistical Method with Information from Satellites, Models, and Monitors. Environ Sci Technol. 2016;50(7):3762–72. Epub 2016/03/10. doi: 10.1021/acs.est.5b05833 . 26953851

[18] Mondini A, Chiaravalloti-Neto F. Spatial correlation of incidence of dengue with socioeconomic, demographic and environmental variables in a Brazilian city. Sci Total Environ. 2008;393(2–3):241–8. Epub 20080208. doi: 10.1016/j.scitotenv.2008.01.010 . 18262225

[19] Ives AR, Zhu J. Statistics for correlated data: phylogenies, space, and time. Ecol Appl. 2006;16(1):20–32. doi: 10.1890/04-0702 . 16705958

[20] Gu J, Shi Y, Zhu Y, Chen N, Wang H, Zhang Z, et al. Ambient air pollution and cause-specific risk of hospital admission in China: A nationwide time-series study. PLoS Med. 2020;17(8):e1003188. Epub 2020/08/08. doi: 10.1371/journal.pmed.1003188 ; PubMed Central PMCID: PMC7410211. 32760064 PMC7410211

[21] Elbarbary M, Honda T, Morgan G, Kelly P, Guo Y, Negin J. Ambient air pollution exposure association with diabetes prevalence and glycosylated hemoglobin (HbA1c) levels in China. Cross-sectional analysis from the WHO study of AGEing and adult health wave 1. J Environ Sci Health A Tox Hazard Subst Environ Eng. 2020;55(10):1149–62. Epub 2020/07/03. doi: 10.1080/10934529.2020.1787011 . 32615056

[22] Jacob AM, Datta M, Kumpatla S, Selvaraj P, Viswanthan V. Prevalence of Diabetes Mellitus and Exposure to Suspended Particulate Matter. J Health Pollut. 2019;9(22):190608. Epub 2019/07/02. doi: 10.5696/2156-9614-9.22.190608 ; PubMed Central PMCID: PMC6555252. 31259084 PMC6555252

[23] Hernandez AM, Gimeno Ruiz de Porras D, Marko D, Whitworth KW. The Association Between PM2.5 and Ozone and the Prevalence of Diabetes Mellitus in the United States, 2002 to 2008. J Occup Environ Med. 2018;60(7):594–602. Epub 2018/04/11. doi: 10.1097/JOM.0000000000001332 . 29634612 PMC8851375

[24] (2013). EPA. National Ambient Air Quality Standards for Particulate Matter. Federal Register 78, 3085–3287.

[25] Xu D, Zhang Y, Sun Q, Wang X, Li T. Long-term PM2.5 exposure and survival among cardiovascular disease patients in Beijing, China. Environ Sci Pollut Res Int. 2021. Epub 2021/04/24. doi: 10.1007/s11356-021-14043-w 33890220

[26] Kim SR, Choi S, Kim K, Chang J, Kim SM, Cho Y, et al. Association of the combined effects of air pollution and changes in physical activity with cardiovascular disease in young adults. Eur Heart J. 2021;42(25):2487–97. Epub 2021/03/30. doi: 10.1093/eurheartj/ehab139 . 33780974

[27] Finger S, Almli CR. Brain damage and neuroplasticity: mechanisms of recovery or development? Brain Res. 1985;357(3):177–86. Epub 1985/12/01. doi: 10.1016/0165-0173(85)90023-2 3913491

[28] Wang M, Zhou T, Song Y, Li X, Ma H, Hu Y, et al. Joint exposure to various ambient air pollutants and incident heart failure: a prospective analysis in UK Biobank. Eur Heart J. 2021;42(16):1582–91. Epub 2021/02/03. doi: 10.1093/eurheartj/ehaa1031 ; PubMed Central PMCID: PMC8060055. 33527989 PMC8060055

[29] Slawsky E, Ward-Caviness CK, Neas L, Devlin RB, Cascio WE, Russell AG, et al. Evaluation of PM2.5 air pollution sources and cardiovascular health. Environ Epidemiol. 2021;5(3):e157. Epub 2021/06/17. doi: 10.1097/EE9.0000000000000157 ; PubMed Central PMCID: PMC8196100. 34131618 PMC8196100

[30] Wang B, Eum KD, Kazemiparkouhi F, Li C, Manjourides J, Pavlu V, et al. The impact of long-term PM2.5 exposure on specific causes of death: exposure-response curves and effect modification among 53 million U.S. Medicare beneficiaries. Environ Health. 2020;19(1):20. Epub 2020/02/19. doi: 10.1186/s12940-020-00575-0 ; PubMed Central PMCID: PMC7026980. 32066433 PMC7026980

[31] Farhadi Z, Abulghasem Gorgi H, Shabaninejad H, Aghajani Delavar M, Torani S. Association between PM2.5 and risk of hospitalization for myocardial infarction: a systematic review and a meta-analysis. BMC Public Health. 2020;20(1):314. Epub 2020/03/14. doi: 10.1186/s12889-020-8262-3 ; PubMed Central PMCID: PMC7068986. 32164596 PMC7068986

