# Supplementary material for: The links of fine airborne particulate matter exposure to occurrence of cardiovascular and metabolic diseases in Michigan, USA
Source: PLOS Glob Public Health. 2022 Aug 5;2(8):e0000707. doi: 10.1371/journal.pgph.0000707 (PMC10021276; doi:10.1371/journal.pgph.0000707)
Supplement: S1 Fig — (DOCX) [file pgph.0000707.s001.docx]

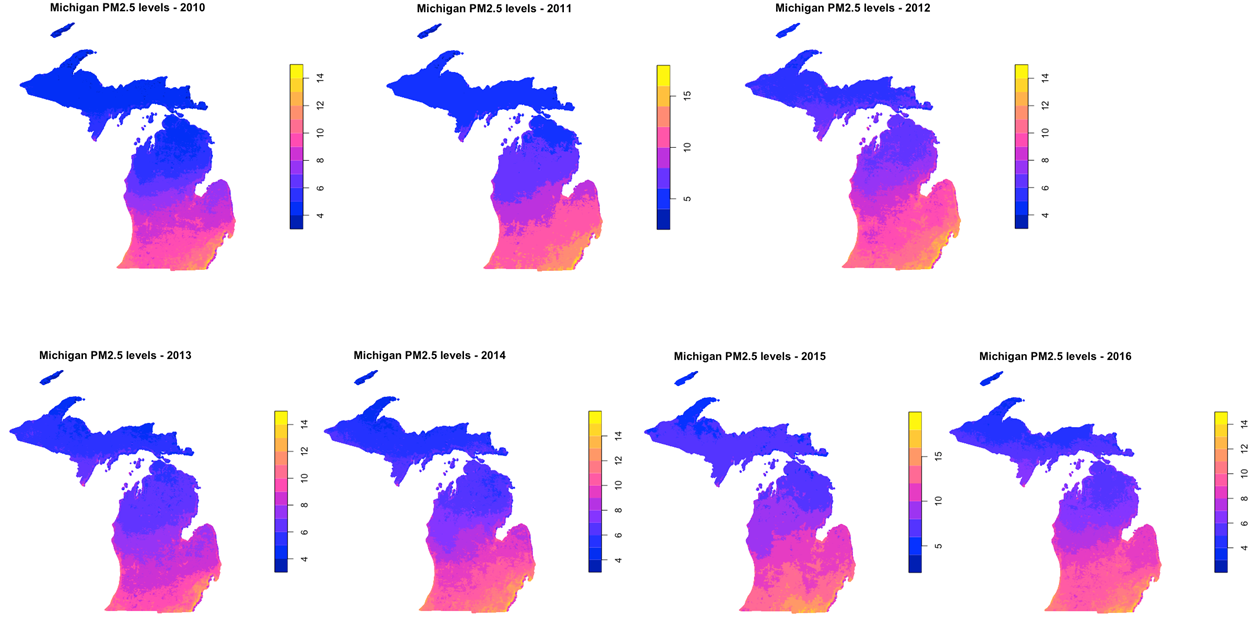


**S1 Fig.** Heat maps showing PM_2.5_ levels across the state of Michigan for years 2010-2016. Annual concentrations of ground-level PM_2.5_ using high-resolution 0.01°x0.01° gridded data retrieved from multiple satellite, surface-level monitors. The non-copyrighted data source at the public domain is provided by the US Census Bureau. The base layer of the map used is publicly available, licensure under “Public domain information”, and it has no constraints to use or access. Link to base layer: <https://hifld-geoplatform.opendata.arcgis.com/datasets/us-county-boundaries/explore?location=28.359536%2C68.376810%2C3.00>.
