# Supplementary material for: The links of fine airborne particulate matter exposure to occurrence of cardiovascular and metabolic diseases in Michigan, USA
Source: PLOS Glob Public Health. 2022 Aug 5;2(8):e0000707. doi: 10.1371/journal.pgph.0000707 (PMC10021276; doi:10.1371/journal.pgph.0000707)
Supplement: S1 Table — (DOCX) [file pgph.0000707.s002.docx]

| **Outcome** | **ICD-10-CM** | **ICD-9-CM** |
| --- | --- | --- |
| Total Cardiovascular Disease | I00-I78 | 390-434, 436-448 |
| Heart Disease | I00-I09, I11, I13, I20-I51 | 390-398, 402, 404, 410-429 |
| Coronary Heart Disease | I20-I25 | 410-414, 429.2 |
| Total Stroke | I60-I69 | 430-434, 436-438 |
| Ischemic Stroke | I63, I65-I66 | 433-434 |
| Hemorrhagic Stroke | I60-I62 | 430-432 |
| Hypertension | I10-I15 | 401-405 |

**S1 Table**. International Classification of Diseases, 9^th^ Revision, Clinical Modification (ICD-9-CM, for hospitalizations before 2015) and ICD-10-CM (for hospitalizations after 2015) codes for cardiovascular and cerebrovascular diseases included in the study.
