# Supplementary material for: The links of fine airborne particulate matter exposure to occurrence of cardiovascular and metabolic diseases in Michigan, USA
Source: PLOS Glob Public Health. 2022 Aug 5;2(8):e0000707. doi: 10.1371/journal.pgph.0000707 (PMC10021276; doi:10.1371/journal.pgph.0000707)
Supplement: S2 Table — (DOCX) [file pgph.0000707.s003.docx]

|  | PM2.5 | DM Prev | DM DR | CVD HR | CVD DR | CHD HR | CHD DR | HD HR | HD DR | Stroke HR | Stroke DR | Isch Str HR | Isch Str DR | Hem Str HR | Hem Str DR | HTN HR | Obesity Prev |
| --- | --- | --- | --- | --- | --- | --- | --- | --- | --- | --- | --- | --- | --- | --- | --- | --- | --- |
| PM_2.5_ | 1.00 | 0.04 | -0.46 | 0.44 | 0.20 | 0.24 | -0.07 | 0.45 | 0.17 | 0.57 | -0.16 | 0.44 | -0.29 | 0.76 | -0.03 | 0.68 | 0.20 |
| Diabetes Prev | 0.04 | 1.00 | 0.33 | 0.30 | 0.41 | 0.29 | 0.28 | 0.29 | 0.39 | 0.16 | 0.18 | 0.19 | 0.11 | 0.00 | 0.20 | 0.11 | 0.52 |
| Diabetes DR | -0.46 | 0.33 | 1.00 | 0.08 | 0.34 | 0.30 | 0.41 | 0.08 | 0.32 | -0.21 | 0.33 | -0.10 | 0.29 | -0.51 | 0.11 | -0.24 | 0.20 |
| CVD HR | 0.44 | 0.30 | 0.08 | 1.00 | 0.60 | 0.89 | 0.45 | 0.99 | 0.62 | 0.67 | -0.01 | 0.65 | -0.29 | 0.45 | 0.17 | 0.72 | 0.40 |
| CVD DR | 0.20 | 0.41 | 0.34 | 0.60 | 1.00 | 0.62 | 0.89 | 0.61 | 0.98 | 0.27 | 0.26 | 0.25 | 0.06 | 0.19 | 0.08 | 0.45 | 0.34 |
| CHD HR | 0.24 | 0.29 | 0.30 | 0.89 | 0.62 | 1.00 | 0.56 | 0.90 | 0.65 | 0.49 | 0.02 | 0.52 | -0.24 | 0.24 | 0.26 | 0.52 | 0.38 |
| CHD DR | -0.07 | 0.28 | 0.41 | 0.45 | 0.89 | 0.56 | 1.00 | 0.47 | 0.92 | 0.07 | 0.12 | 0.06 | 0.00 | 0.01 | 0.02 | 0.25 | 0.25 |
| HD HR | 0.45 | 0.29 | 0.08 | 0.99 | 0.61 | 0.90 | 0.47 | 1.00 | 0.63 | 0.62 | -0.04 | 0.60 | -0.30 | 0.45 | 0.16 | 0.73 | 0.42 |
| HD DR | 0.17 | 0.39 | 0.32 | 0.62 | 0.98 | 0.65 | 0.92 | 0.63 | 1.00 | 0.23 | 0.14 | 0.21 | -0.04 | 0.18 | 0.03 | 0.47 | 0.33 |
| Stroke HR | 0.57 | 0.16 | -0.21 | 0.67 | 0.27 | 0.49 | 0.07 | 0.62 | 0.23 | 1.00 | 0.09 | 0.96 | -0.16 | 0.60 | 0.23 | 0.54 | 0.12 |
| Stroke DR | -0.16 | 0.18 | 0.33 | -0.01 | 0.26 | 0.02 | 0.12 | -0.04 | 0.14 | 0.09 | 1.00 | 0.16 | 0.74 | -0.22 | 0.34 | -0.19 | -0.02 |
| Isch Str HR | 0.44 | 0.19 | -0.10 | 0.65 | 0.25 | 0.52 | 0.06 | 0.60 | 0.21 | 0.96 | 0.16 | 1.00 | -0.11 | 0.45 | 0.25 | 0.44 | 0.10 |
| Isch Str DR | -0.29 | 0.11 | 0.29 | -0.29 | 0.06 | -0.24 | 0.00 | -0.30 | -0.04 | -0.16 | 0.74 | -0.11 | 1.00 | -0.32 | 0.13 | -0.33 | -0.05 |
| Hem Str HR | 0.76 | 0.00 | -0.51 | 0.45 | 0.19 | 0.24 | 0.01 | 0.45 | 0.18 | 0.60 | -0.22 | 0.45 | -0.32 | 1.00 | 0.02 | 0.70 | 0.11 |
| Hem Str DR | -0.03 | 0.20 | 0.11 | 0.17 | 0.08 | 0.26 | 0.02 | 0.16 | 0.03 | 0.23 | 0.34 | 0.25 | 0.13 | 0.02 | 1.00 | 0.01 | 0.09 |
| HTN Hosp | 0.68 | 0.11 | -0.24 | 0.72 | 0.45 | 0.52 | 0.25 | 0.73 | 0.47 | 0.54 | -0.19 | 0.44 | -0.33 | 0.70 | 0.01 | 1.00 | 0.24 |
| Obesity Prev | 0.20 | 0.52 | 0.20 | 0.40 | 0.34 | 0.38 | 0.25 | 0.42 | 0.33 | 0.12 | -0.02 | 0.10 | -0.05 | 0.11 | 0.09 | 0.24 | 1.00 |
| Inactivity | 0.13 | 0.51 | 0.25 | 0.37 | 0.46 | 0.42 | 0.42 | 0.37 | 0.44 | 0.15 | 0.11 | 0.18 | 0.08 | 0.10 | 0.16 | 0.14 | 0.36 |
| Black | 0.45 | 0.10 | -0.36 | -0.01 | 0.02 | -0.22 | -0.13 | -0.01 | -0.03 | 0.18 | 0.07 | 0.06 | 0.08 | 0.43 | 0.04 | 0.31 | -0.04 |
| White | -0.37 | -0.05 | 0.46 | 0.17 | 0.21 | 0.36 | 0.33 | 0.18 | 0.26 | -0.11 | 0.05 | 0.00 | -0.11 | -0.37 | -0.05 | -0.17 | 0.15 |
| Hispanic | 0.76 | -0.05 | -0.52 | 0.16 | -0.02 | -0.03 | -0.25 | 0.16 | -0.07 | 0.42 | -0.14 | 0.32 | -0.11 | 0.64 | -0.01 | 0.44 | 0.02 |
| # Hospitals | 0.50 | -0.08 | -0.34 | 0.01 | 0.05 | -0.14 | -0.04 | 0.03 | 0.05 | 0.14 | -0.23 | 0.02 | -0.23 | 0.45 | -0.03 | 0.36 | -0.13 |
| No Insurance | -0.48 | 0.28 | 0.46 | -0.04 | 0.24 | 0.20 | 0.33 | -0.04 | 0.23 | -0.20 | 0.34 | -0.11 | 0.38 | -0.45 | 0.25 | -0.31 | 0.02 |
| No HS | 0.03 | 0.53 | 0.33 | 0.37 | 0.59 | 0.49 | 0.57 | 0.37 | 0.60 | 0.16 | 0.11 | 0.19 | 0.07 | 0.07 | 0.10 | 0.14 | 0.33 |
| Poverty | -0.16 | 0.40 | 0.38 | 0.21 | 0.56 | 0.23 | 0.55 | 0.21 | 0.54 | 0.00 | 0.21 | 0.03 | 0.13 | -0.08 | 0.12 | 0.12 | 0.20 |
| Med Income | 0.55 | -0.39 | -0.72 | -0.09 | -0.51 | -0.29 | -0.64 | -0.09 | -0.51 | 0.27 | -0.25 | 0.17 | -0.21 | 0.46 | -0.11 | 0.20 | -0.13 |
| Med HomeVal | 0.36 | -0.42 | -0.63 | -0.19 | -0.60 | -0.34 | -0.70 | -0.19 | -0.60 | 0.13 | -0.20 | 0.07 | -0.15 | 0.26 | -0.09 | 0.08 | -0.19 |
| Age Above 65 | -0.65 | 0.11 | 0.63 | -0.11 | 0.08 | 0.18 | 0.23 | -0.12 | 0.09 | -0.34 | 0.20 | -0.20 | 0.19 | -0.57 | 0.12 | -0.44 | -0.06 |
| High Chol | -0.52 | 0.25 | 0.61 | 0.03 | 0.22 | 0.29 | 0.34 | 0.02 | 0.23 | -0.21 | 0.19 | -0.09 | 0.11 | -0.45 | 0.09 | -0.34 | 0.00 |
| Chol Med Nonad | 0.51 | 0.08 | -0.23 | 0.18 | 0.33 | 0.03 | 0.17 | 0.19 | 0.27 | 0.30 | 0.04 | 0.19 | 0.10 | 0.47 | -0.02 | 0.46 | 0.05 |
| BP Med Nonad | 0.55 | 0.34 | -0.11 | 0.58 | 0.49 | 0.46 | 0.25 | 0.58 | 0.44 | 0.54 | 0.09 | 0.48 | -0.11 | 0.57 | 0.26 | 0.68 | 0.23 |
| Current Smokers | -0.12 | 0.60 | 0.39 | 0.28 | 0.57 | 0.38 | 0.58 | 0.28 | 0.57 | 0.08 | 0.20 | 0.14 | 0.19 | -0.12 | 0.03 | 0.00 | 0.36 |

**S2 Table**. Correlation Matrix showing Spearman Correlation Coefficients (𝜌) between all variables. CVD, Total Cardiovascular Disease; CHD, Coronary Heart Disease; HD, Heart Disease (HD); Isch Str, Ischemic Stroke; Hem Str, Hemorrhagic Stroke; HTN, Hypertension. HR = Hospitalization Rate; DR = Death Rate; Prev = Prevalence Rate; Chol = Cholesterol; Med = Medication; Nonad = Nonadherence; BP = Blood Pressure.
